# “Listening to the silence quietly”: investigating the value of cultural immersion and remote experiential learning in preparing midwifery students for clinical practice

**DOI:** 10.1186/1756-0500-7-685

**Published:** 2014-10-02

**Authors:** Rosalie D Thackrah, Sandra C Thompson, Angela Durey

**Affiliations:** Western Australian Centre for Rural Health, University of Western Australia and Adjunct Senior Teaching Fellow, Faculty of Health Sciences, Curtin University, Perth, Western Australia Australia; Faculty of Health Sciences, Curtin University, Perth, Western Australia Australia; School of Dentistry, University of Western Australia and Senior Research Fellow, Faculty of Health Sciences, Curtin University, Perth, Western Australia Australia

**Keywords:** Midwifery education, Aboriginal health, Cultural immersion, Cultural competency

## Abstract

**Background:**

Cultural immersion programs are increasingly offered to medical and health science students in an effort to provide experiential learning opportunities that focus on ‘the self’ as well as ‘the other’. Immersion programs encourage self-reflection on attitudes towards cultural differences, provide opportunities to build relationships and work with community members, and allow students to apply knowledge and skills learned in training programs in a supervised practice setting. The aim of this paper is to describe midwifery students’ reflections on a remote Aboriginal clinical placement that has been offered at a Western Australian university since 2010.

**Methods:**

Interviews were conducted over a period of 15 months with the first seven participants who completed the program. At the time of interview, four participants were in the final year of their undergraduate degree and three were practicing midwives. In addition, access was given to a detailed journal kept by one participant during the placement. Interviews also were conducted with midwifery staff at the university and practice setting, although the focus of this paper is upon the student experience.

**Results:**

Student selection, preparation and learning experiences as well as implications of the placement for midwifery practice are described. The remote clinical placement was highly valued by all students and recommended to others as a profound learning experience. Highlights centred on connections made with community members and cultural knowledge learned experientially, while challenges included geographic and professional isolation and the complexities of health care delivery in remote settings, especially to pregnant and birthing Aboriginal women. All students recognised the transferability of the knowledge and skills acquired to urban settings, and some had already incorporated these learnings into clinical practice.

**Conclusions:**

Cultural immersion programs have the potential to provide students with rich learning experiences that cannot be acquired in classroom settings. In Aboriginal communities on the Ngaanyatjarra Lands students gained valuable insights into the impact of isolation on health service delivery, the extent and strength of cultural traditions in the region, and a heightened awareness of the difficulties encountered by pregnant and birthing Aboriginal women in remote locations.

**Electronic supplementary material:**

The online version of this article (doi:10.1186/1756-0500-7-685) contains supplementary material, which is available to authorized users.

## Background

Mandatory inclusion of Aboriginal and Torres Strait Islander^a^ content in medicine, nursing and midwifery programs in Australian universities and the recommendation of Universities Australia that ‘Indigenous knowledges and perspectives’ are embedded in all university curricula [1], have focused attention on the concept of Indigenous cultural competency. However, issues of how it is reliably acquired, assessed and effectively translated into practice still need attention. The ‘Guiding Principles for Developing Indigenous Cultural Competency in Australian Universities’ report defines cultural competence in Indigenous Australian contexts as ‘student and staff knowledge and understanding of Indigenous Australian cultures, histories and contemporary realities and awareness of Indigenous protocols, combined with the proficiency to engage and work effectively in Indigenous contexts congruent to the expectations of Indigenous Australian peoples’ [1]. In the health care context, enhanced accessibility to and improved satisfaction with services, and ultimately better health outcomes for Aboriginal Australians are desirable outcomes associated with a culturally competent workforce.

Community engagement is identified as one of the five guiding principles of a best practice framework for embedding Indigenous cultural competencies into university programs [1]. Community engagement takes many forms including Aboriginal representation on university advisory committees, invitations to deliver a ‘Welcome to Country’ at significant functions, and involvement of students in Aboriginal community-based organisations. Partnerships with local communities are recognised as the ‘primary foundation for building Indigenous cultural competency in university governance, teaching and learning, research and human resources’ [1].

### Cultural immersion programs: aims and outcomes

Cultural immersion programs have the potential to provide opportunities for community engagement and deliver rich learning experiences for students, while simultaneously offering valuable services to communities [2–6]. Rasmussen [2] described an immersion-style pilot project for 32 volunteer medical students that involved a weekend at an Aboriginal cultural centre in the Grampians in Victoria followed by a tour of Aboriginal community-controlled organisations in Melbourne. The aims of the pilot project focused upon providing opportunities for students to build relationships with a diverse group of Aboriginal people and developing a sense that these relationships ‘are both possible and potentially positive and rewarding’ [2]. At the same time, students were encouraged to reflect on their own cultural backgrounds, consider the influence of past practices on contemporary Aboriginal health status, and recognise the diversity and strength within Aboriginal communities. Evaluation of the pilot project which occurred immediately after the intervention suggested that it was a ‘positive and constructive experience’ for students with many describing it as ‘. . . ‘life-changing’. . . with respect to their attitudes towards Aboriginal people and their culture, and towards their own cultural origins and sense of self’ [2].

Another medical student cultural immersion program had the specific aim of reducing racism in medicine. Crampton et al. [3] described a program for third year students in the rural and remote East Cape region of New Zealand. A week in length and designed in collaboration with a Maori based health care provider, the aim was to provide an immersion experience for students and offer health needs assessments for communities. The program was informed by the principles of cultural safety where the focus was upon ‘. . . potential differences between health providers and patients that have an impact on care’ and aimed ‘. . . to minimize any assault on the patient’s cultural identity’ [3]. The authors noted that ‘. . . students are “inducted” into the Maori world of the East Cape according to a protracted and clear entry protocol’; they slept and ate in *marae* (traditional Maori meeting places) and were cared for by *kaiawhina* (local health care workers). Local communities were compensated for time and costs incurred while hosting their guests [3].

Students’ evaluations of the immersion experience were very positive and reflected a heightened awareness of cultural differences. Nevertheless, the authors drew attention to potential risks associated with immersion programs including the need for delicate management of the relationship with communities in rare cases of inappropriate student behaviour. They recommended that programs provide adequate preparation in advance of such experiences and ensure that learnings are systematically reinforced and built upon. They also noted the superficial nature of the exposure due to the limited time frame [3].

While limited exposure is usually identified as a weakness of immersion programs, Playford and Lines [4] reported that a single week of immersion in a rural community practice made a significant contribution to students’ understanding of primary health care principles. Final year students from a diverse range of health disciplines worked in teams in a small, remote town in Western Australia, and were matched with various ‘at risk’ populations, including Aboriginal people. The immersion exercise which formed part of a ‘Country Week’ practice ‘. . . gave students an idea of how to interact with populations and communities unlike their own’ and ‘. . . embedded students’ understanding and appreciation of the community being a significant partner in healthcare’ [4]. Marked shifts in students’ attitudes towards rural communities were observed, and it was recommended that opportunities for immersion experiences be more readily available to students. Others too have confirmed the value of similar immersion experiences [5].

An innovative immersion program that provided opportunities for medical students’ experiential learning while at the same time addressed a physician human resource need has been operating in remote Indigenous communities in north eastern Canada for over 20 years [6]. The Northern Family Medicine (Norfam) program at Memorial University in Canada was developed after consultation with a range of stakeholders including community leaders and Elders, and aimed to address health disparities and a shortage of physicians in the region. The program began with six month rotations for medical residents and later included medical students who stayed in the region for up to two months at a time. As the program grew and the relationship with communities developed the Elders wish ‘. . . to pass on their knowledge and values to new physicians. . .’ resulted in an invitation to students from Innu Elders to join them on a traditional walk where the students slept in tents with their hosts [6]. It was noted that opportunities for experiential learning occurred while they walked, rested, and prepared and ate food together. ‘During the walk, the trainees received first-hand experience of the positive impact on health and wellbeing with the traditional way of living for Innu, and gain an appreciation of the importance of the land for our Indigenous people’ [6].

This experience in a bitterly cold environment was rated highly by students, as were placements in remote Indigenous communities. The principles of ‘two ways’ learning, a combination of scientific and Indigenous traditional thinking, were enacted in this setting where the locus of power shifted from medical staff and students to the Elders. This role reversal gave students a new appreciation and respect for Innu culture and enhanced sensitivity in their interactions with community members [6, 7].

The importance of experiential learning is emphasised in Kolb’s [8] conceptualisation of the learning cycle where experience, observation and reflection, the formulation of abstract concepts and the testing of these concepts in new situations are viewed as integral to every learning process. Rasmussen [2] noted that immersion-style programs attempt to engage students at the point of ‘experience’, however, care must be taken to ensure that they feel safe and are strongly supported to prepare for this type of learning activity.

### Aboriginal maternal and infant health and maternity service provision

Despite improvements over the last decade, Aboriginal women in Australia continue to have higher maternal mortality rates and twice as many low birth weight babies and perinatal deaths compared with their non-Aboriginal counterparts [9]. Council of Australian Government (COAG) funding provided through the ‘Close the Gap’ policy framework has focused on target areas including to ‘halve the gap in mortality rates for Indigenous children under five within a decade’ [10]. In the area of Maternal and Infant Health, the National Strategic Framework for Aboriginal and Torres Strait Islander Health (2009) identified ‘. . . improved antenatal care provision, alcohol and smoking reduction in pregnancy, reducing the rate of low birth weight babies, reducing the rate of teenage pregnancies and birth, and addressing the causes of maternal mortality and early childhood hospitalisations’ as key indicators for improving the health outcomes for Aboriginal mothers and their babies [11].

While socio-economic factors are clearly implicated in risk factors for Aboriginal women, it has been noted that a lack of culturally appropriate maternity services may also be responsible for adverse pregnancy outcomes [7]. With particular reference to the Ngaanyatjarra women of Western Australia, Simmonds and colleagues (including four local grandmothers) suggested that mainstream services which do not incorporate or recognise traditional beliefs and practices surrounding pregnancy and childbirth fail to meet the needs of many women and may breach cultural norms [7]. The National Maternity Services Plan (2011) recognised the limitations of maternity service provision and had as one of its aims ‘(to) . . . deliver and expand culturally competent maternity care for Aboriginal and Torres Strait Islander people’ [11]. Implicit in this aim is that maternity services should allow for the incorporation of traditional practices and that this is particularly significant in remote communities.

The National Competency Standards for the Midwife require that midwifery practice is culturally safe. This involves incorporating knowledge of cross-cultural and historical factors into practice, respect for differences in cultural meanings and responses to health and maternity care, recognition of the specific needs of Aboriginal and Torres Strait Islander women and their communities, and recognition and respect for customary law [12]. The inclusion of compulsory content on Aboriginal cultures and history in midwifery programs addresses this competency. However, additional opportunities for experiential learning in community settings can reinforce classroom based learning and enhance cross-cultural understandings.

### The setting: Ngaanyatjarra Lands, Western Australia

In this study, final year undergraduate midwifery students and more recently, postgraduate students were invited to apply for a one week clinical practice placement (excluding travel) on the Ngaanyatjarra Lands in Western Australia. The opportunity, which first arose in 2010, resulted from keen interest expressed by a student in a remote placement and subsequent collaboration between midwifery academics and a practicing midwife who had taken up a position at an Aboriginal Community Controlled Health Service on the Lands. A Memorandum of Understanding set out the practice arrangements and scholarships to cover costs incurred by students were accessed through the Australian College of Nursing (ACN). The ACN also paid a supervision payment to the Ngaanyatjarra Health Service, the host organisation. Reserve entry permit applications administered by the Ngaanyatjarra Council were obtained by students prior to the commencement of the clinical placement [13, 14].

The aim of the placement was to provide an opportunity for students to deliver supervised health care in remote, traditionally orientated Aboriginal communities. Learning outcomes related to the enhancement of student knowledge and understanding of Aboriginal health and cultures in the region with particular reference to Aboriginal women’s health issues, development of communication skills, and recognition of the challenges associated with health care delivery in remote settings, including the difficulties women faced upon relocation to large metropolitan and regional hospitals. The clinical placement did not form part of an assessment (it is planned to be in the future), however, students were required to give a presentation on the experience and include their reflections in a submitted portfolio of work. The presentation formed part of an informal debriefing and dissemination exercise [Personal communications, Academic co-ordinator, midwifery program].

**Figure 1:**
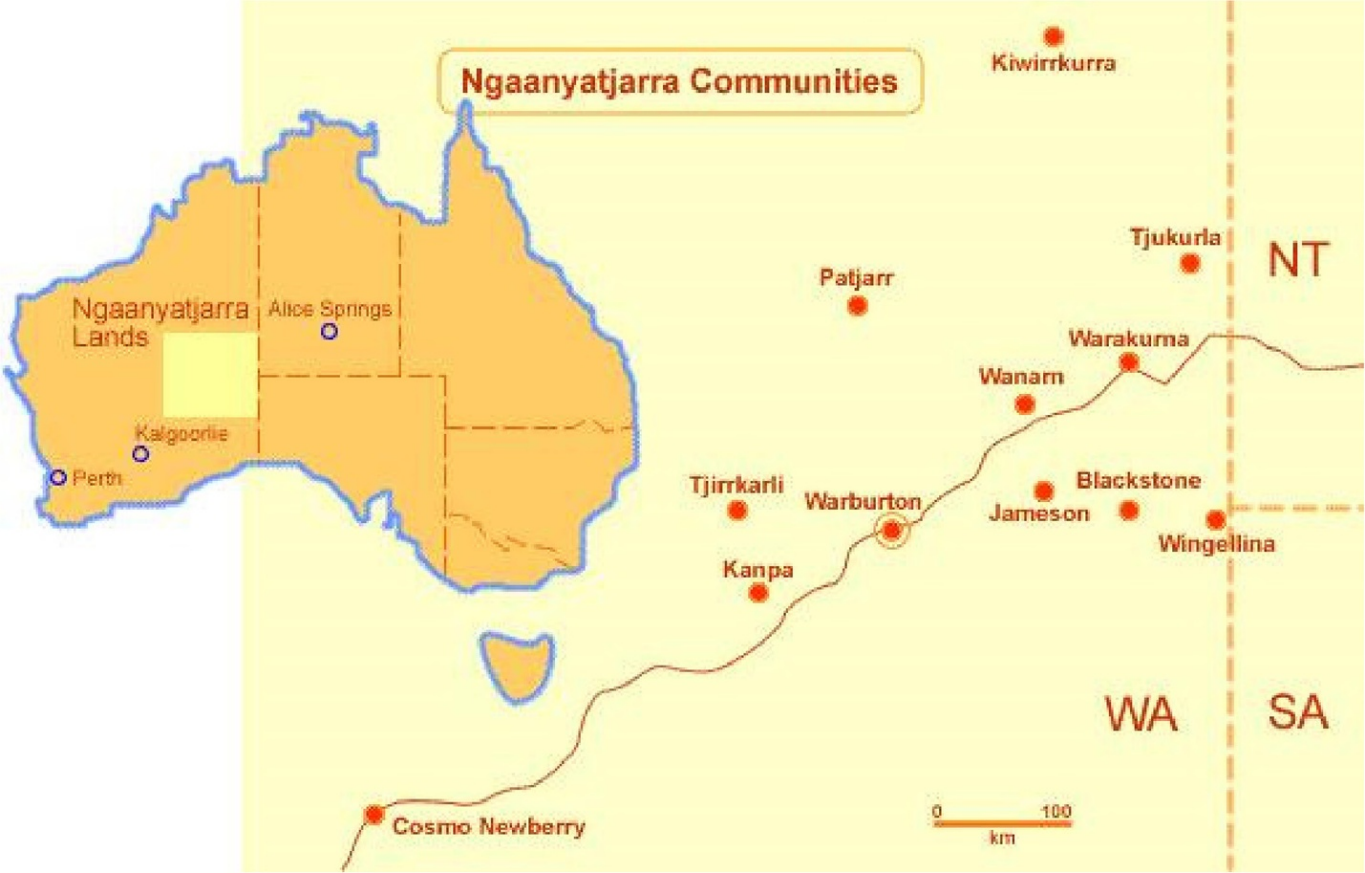
**Ngaanyatjarra communities.** The map of the Ngaanyatjarra Lands identifies the small communities in the region [16]. Permission to use the map has been given by the Ngaanyatjarra Council.

Ngaanyatjarra Lands comprise 250,000 square kilometres in Western Australia and support approximately 2,300 people living in twelve autonomous communities (see Figure 1). Glass and Newberry [15] noted that the Ngaanyatjarra dialect is spoken by around 1,000 people across the region. The communities are ‘dry’ due to local laws prohibiting alcohol, and despite huge changes in lifestyle since the establishment of settled communities Simmonds et al. [7] noted that ‘. . . traditional belief systems and maintenance of the Law remain central to their health and well-being’.

The Ngaanyatjarra Health Service (NHS) is an Aboriginal Community-Controlled Health Service which has its administrative office based in Alice Springs, Northern Territory. Eleven clinics provide primary and preventive health care to people living in widely scattered communities throughout the Lands. The Service employs a multidisciplinary team of health care providers including Aboriginal health workers, registered nurses and midwives [13]. Due to remoteness, pregnant women attend the nearest regional town (Kalgoorlie or Alice Springs) for an ultrasound at 18 weeks and for normal pregnancies are then transferred at around 38 weeks to await the birth of their baby. Antenatal care is provided by a midwife and only occasionally is a baby delivered in the community [7].

This paper, which presents findings on the remote clinical placement offered to midwifery students at a university in Western Australia, adds to our knowledge about the strengths, limitations and risks associated with experiential learning from the perspectives of the student participants.

## Methods

The study population comprised the first seven students who were selected to undertake the Ngaanyatjarra Lands clinical placement between 2010 and 2013. Selection for the placement was based upon a written submission in which students outlined the reasons for their application. Student grades and their capacity to work effectively in a challenging environment, as judged by staff, were also taken into account. The lead researcher, who had previously taught midwifery students (but excluded herself from teaching for the duration of the study), contacted those in the study population by email and telephone. All agreed to participate in a research interview to explore their remote clinical practice experiences. Interviews also were conducted with supervising staff however the focus of this paper is upon the student experience.

One student visited the Ngaanyatjarra Lands in 2010 (the only applicant) and two in each subsequent year, although none visited at the same time. Applications from 2011 always exceeded the placements available. Students were in the final year of their undergraduate program with the exception of one, who applied when the program was opened up to postgraduate diploma students in 2013. All were female, mature-aged students (that is, not straight from school) and three of the seven women had children of their own to make arrangements for while away for up to two weeks.

In-depth, semi-structured, face to face interviews were conducted between November 2012 and February 2014 with six study participants and a phone interview was arranged with one participant who lived in a rural community. Data gathering that utilised in-depth interviews was deemed appropriate for this small-scale, descriptive study which aimed to elicit students’ responses to the experience of delivering health care to Aboriginal women living in remote communities. Participant information and consent forms were provided and written consent obtained prior to the commencement of interviews. Issues related to confidentiality and anonymity were emphasised in the consent form and discussed with students. All gave permission for conversations to be recorded. Interviews, which were conducted by the lead researcher, lasted between 1.5 and 2.5 hours and the recordings were subsequently transcribed. An example of questions used in the interview guide is presented in Additional file 1. Additional probing questions were asked when further information was sought. Although not a requirement, one student kept a journal of the clinical practice experience and made this available to the researcher. Four participants were students at the time of interview and had recently returned from the Lands. Three, who were practicing midwives, recalled the experience retrospectively and were able to comment on the value of the placement to their professional practice.

Patterns of meaning across participant interviews were discerned using standard thematic analysis techniques. Thematic analysis has been described as ‘a method for identifying, analysing and reporting patterns (themes) within the data’ [17]. In this study, transcripts were reviewed multiple times to generate key words and codes were allocated to emerging themes. Marginal notes on the transcripts aided the coding process and provided an audit trail for open scrutiny. Multiple transcript reviews also facilitated the identification of quotations which highlighted emerging patterns of meaning. Early thematic analysis was conducted in the context of existing literature on cultural immersion experiences, and meanings associated with the codes were explored. This process led to further refinement as links between the themes were established and a thematic map was formulated. The interpretative approach described produced the key themes which frame the discussion.

This study which explored the value of cultural immersion and remote experiential learning is part of a broader investigation into culturally secure practice in midwifery education and service provision for Aboriginal women. Ethics approvals were received from the Western Australian Aboriginal Health Ethics Committee and from the Human Research Ethics Committees at the University of Western Australia and Curtin University.

## Results

Initial themes presented in the results include: motivations and preparation; getting there and responding to the setting; encounters: giving and receiving; highlights and challenges; and application to midwifery practice. Three themes emerged from these categories and form the framework for the discussion.

### Motivations and preparation

When asked about motivations to become a midwife, students primarily made reference to a desire to work with women and support them through the journey to motherhood. For some this was a long-standing passion, and all were influenced by a range of factors including past experiences (both personal and work-related), encounters with midwives, insights from literature on early midwifery practice [18, 19] and dissatisfaction with past employment (*‘it was not satisfying my soul’*). The fact that the undergraduate midwifery program could be studied independently of nursing was a strong incentive for those keen to work predominantly with women and their families.

Motivations to undertake the remote clinical placement were varied. Several students were strongly influenced by involvement in constant arguments about Aboriginal issues in social settings.

*This was a really good opportunity for me to go out and . . . have a look and make my own decisions based on what I experienced out there and the people I come into contact with. It was also a perfect opportunity to consolidate what we had actually learnt in our Indigenous studies.*

Another student put it this way:

*I suppose the biggest reason was that I wanted to see it for myself. I wanted to see how an Aboriginal community is, what it looks like, what it feels like, what resources are there, to see the way they live, rather than go by the Indigenous unit, the media, the newspapers, my husband’s point of view, my friend’s point of view. I wanted to see firsthand what it was like, what it was really like.*

Other motivations included seeing how health services operated in remote settings, to develop pre-existing interests and build upon current knowledge in Aboriginal health, to fulfil travel aspirations and enhance job prospects. Several students, who had prior experience working with or living in close proximity to Aboriginal families in rural areas, recalled the impact of these experiences on the development of their attitudes. One, a former lawyer, had travelled to the remote Kimberley region with a Supreme Court judge who presided over criminal trials. She was exposed to the complexities surrounding cultural evidence and the impact on families and communities of high incarceration rates. Another student had witnessed, as a child, discrimination experienced by Aboriginal people in rural areas and only in adulthood had come to understand the policy frameworks that allowed such discrimination to occur. The remote clinical placement was viewed by all students as an opportunity to gain valuable experience and a deeper understanding of Aboriginal health and cultures.

Although preparation for the Ngaanyatjarra Lands clinical placement varied, most students had successfully completed a compulsory core unit on Indigenous health and cultures in the first year of their degree (one student was exempt). The unit, which was designed and delivered by Aboriginal and non-Aboriginal staff was well received by students and all considered it to be important content. One student noted *‘I think (the unit) was valuable. Sometimes it is only when you look back on what you learnt and you are applying it that you realise how valuable it is’*. The Royal College of Nursing scholarship that covered costs associated with the placement required students to complete an *Online Cultural Orientation Training for Health Professionals* offered by the Western Australian Centre for Rural Health, University of Western Australia (http://www.wacrh.uwa.edu.au). All considered this to be a very useful resource. Additional preparation was conducted independently by some students and included reading Ngaanyatjarra Council publications, academic articles and books specific to the region (with a focus on health, culture and language) and discussions with midwives who had worked remotely. An older student commented that it was her prior experiences and reading that had informed her about Aboriginal issues, and in particular, Kim Scott’s book *Benang*[20]. *‘I think that was the most educational book on the treatment of Aboriginals that I have ever read. It really, really shocked me’.* Most students thought that they had prepared diligently for the placement, although in hindsight recognised that there were some situations for which one cannot prepare and require context for the learning to take place.

### Getting there and responding to the setting

The remoteness of the setting took many students by surprise. Getting there involved a flight from Perth to Kalgoorlie or Alice Springs, and then a light plane to a community on the Ngaanyatjarra Lands. The second flight could take up to three hours and was a frightening experience for some.

*I ended up being on the Lands for only one week because it took a couple of days to get there. I had to fly in to Kalgoorlie first, and then stay overnight, and then fly out in the morning in a 12 seater. . . and it was pretty rough, I think it was the worst flight I’ve ever been on. . . everyone was just sort of looking at each other thinking any second now we are all going to die together. I can’t even imagine what it would be like getting on a flight like that at 36 or 37 weeks pregnant, especially if it is your first baby, and it could be your first time on a plane, and it was like that (rough), and you are going to a place you don’t know, on your own. It would be just horrible.*

The isolation and the cultural divide between local community members and white professional staff was also a shock for some students.

*I don’t think I fully realised how isolated some Indigenous communities really are and that you are not really part of the community. Before I went I thought it would be like a small community at a camping ground or caravan park where everyone lives and gets on together, but it was not like that at all.*

One student revelled in the isolation and the setting. *‘ I knew I would like it but did not realise that I would fall in love with it’,* but for others, coming to terms with the isolation and the enormous amount of time spent on the road travelling between communities was difficult, especially when they assessed their productivity. There was a sense of ‘time wasted’ due the vast distances that had to be traversed, although over the duration of the placement, most adjusted to the inevitability of working this way.

With reference to the cultural divide, there had been an expectation on the part of students that they would mix socially with community members but the reality was that there were very few opportunities to develop friendships. One health professional who had been in the region for nearly three years noted *‘no, it doesn’t seem to happen. We are different from them; we are wadjalas’ (white people).* Interactions were strictly professional, and although instances of social interaction were cited, these were very occasional, the exception rather than the rule.

### Encounters: giving and receiving

The rhythm of a typical day for health professionals on the Lands varied and depended upon the community in which they were based. In the small community of Warakurna (population approximately 194 people) situated close to the Western Australian/Northern Territory border, the local midwife and accompanying student packed the car with all necessary equipment and drove to even smaller communities. They were based at the health clinics and worked as a team with the local nurse. The student commented,

*I found it really interesting that if the nurses wanted someone to be seen, they would just get in the ambulance and go to the person’s house and say “Come on, come to the clinic, we want the midwife to see you”. So they knew where everyone lived. Or if someone wasn’t around, they would just ask someone walking by, “Oh, where is such and such” and they would usually know where that person was, “Oh, she’s at Jo’s house”. A lot of the time people didn’t want to come to the clinic.*

Another student explained a typical day this way:

*We would pile into the car . . . and drive to Papulankutja (Blackstone) and the nurse at the clinic would spread the word, the minymaku sisters (‘belonging to women’) were here . . . and women would just slowly filter in. They would have a chat for a while and often not get to the point of what they were in for . . . finally they would often whisper that they had come in for a health check.*

At the clinic, pregnancies were monitored but as there were so few in such small communities, midwives (and students) were involved in many other health-related activities including health promotion, sexual health, arranging breast screening and ultra-sounds, post-natal care, Pap smears and contraception advice. *‘We were doing mostly pap smears, different vaginal swabs, talking about contraception . . . and then there were some women that talked about fertility problems’*. It was during these encounters with women and young teenage girls, that students who were delivering health care services also became recipients of valuable cultural knowledge passed on by patients and colleagues.

A student’s journal entry described a complex situation confronting a young teenage girl who came to a clinic for contraception. She had been in an abusive relationship and had broken bones and burns from a beating which were slowly healing. Her mother-in-law (an esteemed Elder) took the two children away from her while her man was in jail and the girl was missing them. The student started looking at the situation from a family law point of view but colleagues quickly reminded her that the community would turn their back on this girl if she did not follow local protocols. These involved ‘biding her time’ until the mother-in-law decided it was too much for her to look after these children as well as the numerous other grandchildren in her care. ‘*That is what she was hanging around for. She still wants her community. She doesn’t belong anywhere else. That is all she has got’.*

All students commented that anything could happen in a typical day. They observed the attributes of independence and flexibility that enabled health care professionals to work successfully in remote settings, and absorbed cultural learnings that could never be acquired from textbooks. The students gained insights into the complexity of people’s lives, the cultural obligations that must be met and the extraordinary creativity that can emerge within remote, disadvantaged communities and harsh landscapes. Difficulties emerged, however, in reconciling the contradictions in opportunities and realities of community members’ lives. One student described treating a man for burns on his buttocks that occurred when he sat on a fire. He was extremely polite when treated but it subsequently emerged that a few days earlier he had been associated with a violent incident in the community. The following year, the student was surprised to see one of his paintings on the wall of a major art gallery.

During encounters with patients, students also listened as staff incorporated local language into their conversations and observed how this gesture enhanced communication and trust. Most students acknowledged that they received more than they gave.

### Highlights and challenges

Highlights of the remote clinical placement arose out of connections made with Aboriginal people, particularly women and children. Students described opportunistic encounters that provided insights into traditional Aboriginal beliefs and practices, including painting and weaving and birthing stories. Some were fortunate to observe artists at work.

*We were driving around with the Aboriginal health worker trying to find some women but they weren’t there. We went to one house and there were two old ladies. It was a neat-as-a-pin yard . . . on the veranda there was a fire pit with two beds neatly arranged, and these ladies were sitting around a camp fire with all the dogs. The puppies were crawling through, you know, and they were making little sticks, little musical sticks, and one of them was burning a coat hanger in the coals and then she would mark the sticks, And so we said, ‘Oh, can we sit down and have a yack’ and they said ‘Yeah’, and so we sat there and it was just amazing. I will remember it for the rest of my life. I am tearing up just thinking about it. And they were telling me about the seven sisters (stars) and the painting she was doing and we just sat in the dirt and listened to them. It was just such a profound experience for me . . . you can’t learn about it (in the classroom).*

Another student commented on her interactions with children at a sexual health fair where workers were handing out pamphlets. She assisted in a face painting stall and became very popular because she knew how to do Spider Man and butterflies and *‘all the kids were going around with little Spider Man faces, but then you had all the head lice dangling down and getting stuck in the face paint!’* Time spent with an elderly woman who was a birth assistant when she was young was the highlight for one student. The woman in her seventies intentionally came to the clinic and waited as she had heard that a midwifery student was there. She talked about traditional birthing practices on the Lands, special foods, smoking ceremonies conducted to strengthen the mother and baby following birth, and making the birthing area safe. *‘This all happened about 30–40 years ago, and I thought she would like it to still be like that but she was really appreciative of the fact that women now go to Kalgoorlie. . . .’* While smoking ceremonies are still practiced, women are routinely flown to hospitals in large towns for delivery.

Challenges identified by students included witnessing the devastation caused by chronic health problems, feelings of helplessness about effecting change, the large number of scary and mangy dogs, *‘I rocked up one time to this house and there were lots of dogs and I started counting them, and there were 13’,* cultural differences with respect to raising children, the presence of racism among some health workers (evidenced by disparaging comments about community members), and the long evenings and weekends with very little to do and nowhere to go. Despite these challenges, all valued the experience highly, and had recommended the opportunity to other students.

### Application to midwifery practice

All students considered that the learning experiences acquired on the Ngaanyatjarra Lands were transferable to urban settings and those who were practicing midwives provided examples of how they applied knowledge gained in everyday situations. One midwife described a 16 year girl in labour at a large metropolitan hospital in Perth who had flown in from a remote community. She established that she was comfortable, and her mother was with her, and *‘I think I stepped back a little more than I would have if I hadn’t had that remote experience. . . I spent nearly an hour just being quietly with her and that was enough for her, no chatty conversations’.* Another student described how she spent time with a woman from the Kimberley’s who had a mitral valve problem due to rheumatic fever. She went with her in a taxi to another hospital to have an ultrasound on her heart. The cardiologist asked her if she drank alcohol and she said no. The student knew from her notes that she had 10 tinnies a day when she was in town. *‘I had to pipe up and say something. I said, oh xx, is it alright if I say something? I believe you have a few tinnies just when you are in town and she said, oh yeah, I do actually’.* The cardiologist then changed the valve he was going to use because it was incompatible with her alcohol usage. The student realised that the knowledge gained on the Lands, particularly about direct questioning and the desire to please, helped avert a serious problem arising.

Other students commented on insights gained about the vast distances travelled by women to deliver, the huge contrast in settings, and the frightening nature of hospitals, especially if women had travelled to the city on their own. Occasionally connections were made with community members from the Lands.

*One lady I met was in a wheelchair. She’d had a stroke when she was 17, not long after she had had her first baby. (Out on the Lands) we did part of her antenatal care for her next pregnancy . . . and she ended up coming to xx hospital (in Perth) to have her baby (when I was there). I met her when she came in with one of her sisters. I remembered her and gave her a photo of her family that I had taken out in the community, and she pinned that picture up on the board because she was really missing her family. Her baby was born at 33 weeks and I went to visit them both when they were moved to another hospital. Just to have someone say, “Oh, I’ve been to the Lands and do you know such and such” and they say, “Oh yeah, I know that girl, sister”. Making that connection really helps establish trust.*

While most students acknowledged that they may never work remotely, they all cited examples of how their remote clinical placement had improved their midwifery practice and their interactions with all Aboriginal women encountered. *‘I find I tend to seek Aboriginal women out more because I feel their vulnerability much more than I did before’.* While some of the students returned from the Lands with feelings of frustration and helplessness with regards to improving Aboriginal health, especially for women and their babies, nonetheless the experience provided insights into the complexity of the issues involved, the prevalence and richness of cultural traditions maintained in the region and a heightened awareness of the enormous transition required of women who are relocated from the remote Lands to towns and cities to birth.

## Discussion

The aims of cultural immersion programs and the learning outcomes for students are influenced by many factors including the setting, partnership arrangements with communities, student preparation, length of placement, availability of clinical supervision, longevity of the program, opportunities for interaction and reciprocity, and degree of cultural differences encountered [2–6]. The midwifery remote clinical placement described in this paper offered students the opportunity to practice under supervision in very remote, traditionally-oriented Aboriginal communities within the Ngaanyatjarra Lands, Western Australia. The placement which was formalised in 2010 remains in the early phase of development although it is anticipated that more places will become available in future years and the length of the placement will increase from one to two weeks. Limiting factors relate to the availability of accommodation and the additional workload for the supervising midwife [Personal communications, Academic co-ordinator, midwifery program].

Key themes centred on the impact of geographic and professional isolation on midwifery practice; the significance of cultural protocols particularly with respect to communication, and the profound nature and relevance of the learning experience.

### Geographic and professional isolation

The remoteness of the setting and health service delivery which required interminable hours of travel to very small communities widely scattered throughout the Ngaanyatjarra Lands, shocked many students - despite their thorough preparation for the placement. While their stay was brief, all were cognisant of the impact of isolation on pregnant women and their families and gained a deeper understanding of the emotional turmoil that surrounds transfer to regional or tertiary obstetric hospitals for birthing. Simmonds et al. [21] highlighted these issues in their study of the antenatal care and birthing needs of Ngaanyatjarra women. In-depth interviews with 36 women revealed a desire for a support person at antenatal appointments and when transferred to towns to await the birth, though not necessarily to be present at the delivery. Kildea [22] drew attention to the prohibitive costs associated with a family member accompanying women relocating for birth and called for appropriate resourcing to address the disadvantage suffered by remote, pregnant Aboriginal women.

Geographic isolation was also closely associated with professional isolation and this strongly influenced the nature of midwifery and nursing practice. Students observed professional staff taking on higher degrees of responsibility than in urban settings due to the absence of resident doctors, and recognised that characteristics including independence and flexibility were required to survive in remote settings. Cramer [23] used the term ‘amorphous practice’ to describe ‘the changing and inconsistent nature of practice’ with respect to nursing in remote communities. Amorphous practice included a degree of detachment from organisational systems due to isolation, and deviations from the official scope of practice such as assuming some medical roles. Stress associated with over-stepping professional boundaries and ‘. . . the unrelenting demands to serve others’ were linked with fatigue and low morale [23]. Inherent risks are evident in this type of practice for both providers and recipients, and students were acutely aware of the weight of responsibility and high turn-over of staff in remote locations. Their supervising midwife was considered a long term resident; she had stayed for two and a half years before relocating to a less remote setting.

### Significance of cultural protocols

Thorough preparation prior to the clinical placement ensured that students were conversant with cultural protocols surrounding ‘women’s business’ and interactions within the broader community. Nevertheless, they expressed surprise at the widespread use of local languages (mostly Ngaanyatjarra) by community members and the strategic use of ‘language’ by health professionals. Students themselves learned a glossary of terms which facilitated interactions and in the process discovered the significance of such small but important gestures in relationship building.

The role of silence in communication was less easily accommodated by students despite their awareness of its importance. On women’s verandas, conversations between the students and Aboriginal women about their paintings occurred without difficulty, but in health care settings where different dynamics existed and sensitive issues were discussed, communication was far more hesitant. In a paper on ‘conversational silence’ in remote Aboriginal communities, Mushin and Gardner [24] confirmed earlier findings that remote Aboriginal people are more comfortable with longer silences compared with Anglo-Australians, and ‘treat such silences as ordinary’. A number of explanations were offered including less orientation to clock time, more time to interact, proximity and ‘. . . an expectation that there are open ended opportunities to continue a conversation’ [24]. Students in this study identified numerous occasions where they were initially uncomfortable with long silences, but ultimately made connections by ‘listening to the silence quietly’. Given time, women and girls in clinic waiting rooms sometimes (but not always) raised the ‘women’s business’ issues that had brought them there in the first place, but this was usually done indirectly and in a faint whisper, highlighting the sensitive and embarrassing nature of such conversations. The students felt they became better communicators and more culturally secure practitioners as a result of this experiential learning and some subsequently applied these skills in large urban and rural settings.

The significance of cultural protocols also was reinforced when students were made aware of ‘sorry business’ and ‘sorry business’ camps (protocols surrounding death and dying), and conducted health promotion activities. They recognised that their role as visitors in the community did not give them a right to interfere with culturally prescribed patterns of behaviour such as the relationship between a mother -in-law and her grandchildren or the importance attached to Elders in the community. More specifically they became acquainted with cultural sensitivities surrounding *‘minymaku kutju tjukurpa’* (women’s secret/sacred business) as they shadowed the local supervising midwife [Personal communications, (past) Co-ordinator, Maternal and Women’s Health Program, Ngaanyatjarra Health Service].

Midwifery researchers and practitioners with extensive experience in remote Aboriginal communities have suggested adapting western biomedical approaches to pregnancy and birthing to incorporate cultural knowledge and protocols [7, 11, 21, 22, 25, 26]. Simmonds et al. [7] commented that ‘. . . older women (in their study) lamented the lack of opportunity for passing on knowledge to younger women . . . and (indicated) that many young women do not listen to their grandmothers anymore’. It is interesting to note that in this study one grandmother came to a clinic specifically to talk with a midwifery student about traditional birthing practices. For the student this conversation was the highlight of the practice experience; for the grandmother, it appeared to be an opportunity to disseminate information of importance to her and the wider community to an appreciative audience. Despite concerns expressed by older women about loss of traditional knowledge around birthing and the desire for opportunities to maintain these practices, most acknowledged the benefits of western medicine and the desirability of relocation for birthing, albeit with a support person [7, 21].

With respect to cultural protocols students quickly learned that opportunities for social interactions with community members were limited. Not only was language a barrier, but maintenance of very traditional lifestyles on the Lands, meant the presence of an extensive social and cultural divide. This contrasted with other cultural immersion programs [3, 6] but does not preclude the possibility that over time, and with more time, interactions may become less inhibited. When rare opportunities for social interaction did arise, these were identified as highlights of the placement.

While the focus of this study was upon midwifery students’ perceptions of the cultural immersion experience, it is recognised that the views of Aboriginal women, as recipients of the health services delivered by the students, would have added further richness to the data collected. Nevertheless, rapport and respect were the hallmarks of student interactions with Aboriginal women and as has been noted, women used these interactions as an opportunity to teach students about traditional cultural practices relating to *minymaku kutja tjukurpa.* This ‘two ways’ learning would not have occurred if students had been insensitive to their surroundings.

### Profound and relevant learning experience

Extensive preparation, community involvement, voluntary participation and strong student support in the field are factors associated with successful student cultural immersion experiences [2, 3, 6]. Potential risks may arise if students are challenged or confronted by unfamiliar and /or unsafe situations, or where they themselves cause offense to community members. Extensive interviews revealed that while feelings of helplessness and frustration were occasionally experienced by students with respect to the extent of health problems on the Lands, all participants in the clinical placement considered the learning experience to be profound and relevant to their midwifery practice, regardless of the setting.

Experiential learning that occurred was frequently referred to as learning that cannot be acquired from text books or lectures. Paul et al’s study [27] of medical student reflections following the completion of a case history of an Aboriginal patient reported similar sentiments expressed. Some students commented that they ‘learned a lot more than textbooks could ever teach’ and that the exercise was ‘beyond books!’ For others, it was their ‘first in depth interaction with an Indigenous patient’ [27]. Prout et al. [5] also reported on the transformative potential of facilitated learning ‘in situ’, especially where interactions with Aboriginal people were encouraged. Although midwifery students spent only one week on the Ngaanyatjarra Lands, they were keen to emphasise the positive and unforgettable nature of the experience and recommend it to others, especially younger students who were more reluctant to apply for the placement.

## Conclusions

Cultural immersion programs which provide opportunities for students to learn from and interact with community members in supervised practice settings, have the potential to deliver rich learning experiences that cannot be acquired in classroom settings. In a clinical placement on the Ngaanyatjarra Lands, Western Australia, midwifery students gained valuable insights into the impact of isolation on health service delivery, the complexity of the issues involved in delivering care, the extent and strength of Aboriginal cultural traditions in the region, and a heightened awareness of the difficulties encountered by pregnant and birthing Aboriginal women in remote locations.

All participants valued the placement highly and identified profound learning experiences that arose out of their interactions with Aboriginal community members and health professionals on the Lands. While most acknowledged that they may never work remotely, they recognised the transferability of their learnings to urban and rural settings. By their own assessment, they had applied knowledge and skills acquired on the Lands in their clinical practice and progressed along the Indigenous cultural competency continuum, although most were acutely aware of how little they really knew.

## Endnote

^a^In the Australian context, the term ‘Aboriginal and Torres Strait Islander’ is frequently abbreviated to ‘Aboriginal’ and that is the usage in this paper. The term ‘Indigenous’, which also refers to Aboriginal and Torres Strait Islanders, appears in this paper when reference is made to literature using the term or when it is used in quotations. These terms are often used interchangeably by government bodies, the academy and by Aboriginal people themselves and hence it is not possible to impose consistency.

## Authors’ information

RDT is a PhD candidate, Western Australian Centre for Rural Health, University of Western Australia and Adjunct Senior Teaching Fellow, Faculty of Health Sciences, Curtin University,Western Australia.

SCT is Director and Professor of Rural Health, Western Australian Centre for Rural Health, University of Western Australia.

AD is Research Associate Professor in the School of Dentistry, University of Western Australia and Senior Research Fellow, Faculty of Health Sciences, Curtin University, Western Australia.

## Electronic supplementary material

Additional file 1: **Questions from the interview guide.** (DOCX 17 KB)

## References

[1] Universities Australia (2011). Guiding Principles for Developing Indigenous Cultural Competency in Australian Universities.

[2] Rasmussen L (2001). Towards Reconciliation in Aboriginal Health: Initiatives for Teaching Medical Students about Aboriginal Issues.

[3] Crampton P, Dowell A, Parkin C, Thompson C (2003). Combatting Effects of Racism Through a Cultural Immersion Medical Education Program. Acad Med.

[4] Playford D, Lines A (2013). Diminishing the distance between patients and providers: The impact of rural community immersion on students' appreciation of primary health care. Focus Health Prof Educ.

[5] Prout S, Lin I, Nattabi B, Green C (2013). 'I could never have learned this in a lecture': transformative learning in rural health education. Adv in Health Sci Educ.

[6] Jong M (2011). Learning about Indigenous health immersion and living with Elders in northern Canada. Focus Health Prof Educ.

[7] Simmonds D, West L, Porter J, Tangey A, Davies M, O’Rourke P, Holland C (2010). A 'Two Ways' Approach to Improving Antenatal Education for Ngaanyatjarra Women. Aborig Isl Health Work J.

[8] Kolb D (1984). Experiential learning: Experience as the source of learning and development.

[9] Department of Families, Housing, Community Services and Indigenous Affairs (2013). Closing the Gap Prime Minister's Report.

[10] Department of Families, Housing, Community Services and Indigenous Affairs (2009). Closing the Gap on Indigenous Disadvantage: The Challenge for Australia.

[11] Kildea S, Van Wagner V (2012). 'Birthing on Country' maternity service delivery models: a rapid review.

[12] Australian Nursing and Midwifery Council (2006). National Competency Standards for the Midwife.

[13] Ngaanyatjarra Health Service: Alice Springs, Northern Territoryhttp://www.nghealth.org.au

[14] Ngaanyatjarra Council: Alice Springs, Northern Territoryhttp://www.ngaanyatjarra.org.au

[15] Glass A, Newberry B (2005). Ngaanyatjarra Word List (Rev Ed).

[16] Ngaanyatjarra Health Service: *Orientation Information for Healthcare Professionals Visiting the Ngaanyatjarra Lands*. Alice Springs: Northern Territory; no date. http://www.nghealth.org.au

[17] Liamputtong P (2011). Qualitative Research Methods.

[18] Brooks G (2001). Year of Wonders: a novel of the plague.

[19] Diamant A (1997). The red tent.

[20] Scott K (1999). Benang – From the Heart.

[21] Simmonds D, West L, Porter J, Davies M, Holland C, Preston-Thomas A, O'Rourke P, Tangey A (2012). The role of support person for Ngaanyatjarra women during pregnancy and birth. Women Birth.

[22] Kildea S (2006). Risky business: contested knowledge over safe birthing services for Aboriginal women. Health Sociol Rev.

[23] Cramer J (2006). Amorphous Practice: Nursing in a remote Indigenous community of Australia. Contemp Nurse.

[24] Mushin I, Gardner R (2009). Silence is talk: Conversational silence in Australian Aboriginal talk-in-interaction. J Pragmat.

[25] Kildea S, Wardaguga M, Selin H, Stone P (2009). Childbirth in Australia: Aboriginal and Torres Strait Islander Women. Childbirth across cultures: ideas and practices of pregnancy, childbirth and the postpartum.

[26] Kruske S, Kildea S, Barclay L (2006). Cultural safety and maternity care for Aboriginal and Torres Strait Islander Australians. Women Birth.

[27] Paul D, Allen C, Edgill P (2011). Turning the corner. Assessment: a key strategy to engagement and understanding in Indigenous health. Focus Health Prof Educ.

