# Supplementary material for: “Listening to the silence quietly”: investigating the value of cultural immersion and remote experiential learning in preparing midwifery students for clinical practice
Source: BMC Res Notes. 2014 Oct 2;7:685. doi: 10.1186/1756-0500-7-685 (PMC4283110; doi:10.1186/1756-0500-7-685)
Supplement: Supplementary file 1 — Additional file 1: Questions from the interview guide. (DOCX 17 KB) [file 13104_2014_3394_MOESM1_ESM.docx]

**Additional file 1**

1. Can you tell me what motivated you to undertake the midwifery program?
2. In your first year you completed the Indigenous health and culture unit. Can you tell me about the impact this unit had upon you? What key learnings did you take from it?
3. Was Indigenous content raised in other units completed in your program? If so, what form did it take?
4. Why did you apply to go on a remote placement for your final clinical prac?
5. Did you receive any financial assistance?
6. How well prepared were you for the experience?
7. Can you tell me about the setting, how you got there, what your feelings were when you arrived?
8. What was the nature of your role and what sort of clinical experience did you receive? Were you happy with the support you received from the staff you worked with?
9. Can you describe a typical working day for me?
10. Were the clinics well resourced? Can you describe clinics you visited?
11. What about the evenings and weekends? How did you spend them?
12. Did you have much social contact with community members apart from in the working environment?
13. What were the best things that came out of this clinical experience for you personally and professionally?
14. Did you encounter any challenges?
15. Would you recommend this clinical practice to other students as a good learning experience?
16. From your experience what more needs to be done to improve interactions between health professionals and Aboriginal women?
